# Possible impacts of a future grand solar minimum on climate: Stratospheric and global circulation changes

**DOI:** 10.1002/2014JD022022

**Published:** 2015-09-17

**Authors:** A. C. Maycock, S. Ineson, L. J. Gray, A. A. Scaife, J. A. Anstey, M. Lockwood, N. Butchart, S. C. Hardiman, D. M. Mitchell, S. M. Osprey

**Affiliations:** ^1^ Centre for Atmospheric ScienceUniversity of CambridgeCambridgeUK; ^2^ National Centre for Atmospheric ScienceUK; ^3^ Met Office Hadley CentreMet OfficeExeterUK; ^4^ Department of Atmosphere, Ocean and Planetary PhysicsUniversity of OxfordOxfordUK; ^5^ Department of MeteorologyUniversity of ReadingReadingUK

**Keywords:** grand solar minimum, solar influences on climate, stratosphere‐troposphere coupling

## Abstract

It has been suggested that the Sun may evolve into a period of lower activity over the 21st century. This study examines the potential climate impacts of the onset of an extreme “Maunder Minimum‐like” grand solar minimum using a comprehensive global climate model. Over the second half of the 21st century, the scenario assumes a decrease in total solar irradiance of 0.12% compared to a reference Representative Concentration Pathway 8.5 experiment. The decrease in solar irradiance cools the stratopause (∼1 hPa) in the annual and global mean by 1.2 K. The impact on global mean near‐surface temperature is small (∼−0.1 K), but larger changes in regional climate occur during the stratospheric dynamically active seasons. In Northern Hemisphere wintertime, there is a weakening of the stratospheric westerly jet by up to ∼3–4 m s^−1^, with the largest changes occurring in January–February. This is accompanied by a deepening of the Aleutian Low at the surface and an increase in blocking over Northern Europe and the North Pacific. There is also an equatorward shift in the Southern Hemisphere midlatitude eddy‐driven jet in austral spring. The occurrence of an amplified regional response during winter and spring suggests a contribution from a top‐down pathway for solar‐climate coupling; this is tested using an experiment in which ultraviolet (200–320 nm) radiation is decreased in isolation of other changes. The results show that a large decline in solar activity over the 21st century could have important impacts on the stratosphere and regional surface climate.

## Key Points

- A future decline in solar activity would not offset projected global warming
- A future decline in solar activity could have larger regional effects in winter
- Top‐down mechanism contributes to Northern Hemisphere regional response

## 1 Introduction

Electromagnetic radiation from the Sun is a fundamental source of energy for the terrestrial climate system. Therefore, changes in solar activity have the potential to influence global climate. The Sun's output varies on a number of characteristic time scales. In the context of Earth's climate, the most frequently studied of these is the approximately 11 year (Schwabe) solar cycle, which is typically associated with a maximum to minimum change in total solar irradiance (TSI) of ∼1 W m^−2^ or ∼0.07% [*Gray et al.*, 2010]. The Sun's output is also known to vary on longer time scales; however, characterizing these variations requires a much longer record of solar activity. Direct measurements of sunspot numbers extend back to 1610 [*Ribes and Nesme‐Ribes*, 1993], but proxy records must be used to reconstruct solar activity further back in time. *Steinhilber et al.* [2008] compiled a record of the solar modulation potential, *ϕ*, for the last 9300 years. This is a measure of the shielding of the Earth from galactic cosmic rays by the Sun's magnetic field and is derived from cosmogenic radionuclide data from ice cores. *Lockwood* [2010] showed that when smoothed to remove the signal of the 11 year cycle, the *ϕ* record exhibits “grand maxima” and “grand minima” with a time scale of ∼100–200 years. The period of relatively high solar activity over the last ∼50 years has coincided with a so‐called “grand solar maximum,” and the period of low solar activity during the late seventeenth century, known as the Maunder Minimum (MM), is believed to have coincided with a “grand solar minimum.”

*Abreu et al.* [2008] conducted a statistical analysis of the *ϕ* record and deduced that the current grand maximum is only likely to persist for up to another 15–36 years, after which the Sun would be expected to evolve toward a state of lower output. It has been suggested that the amplitude and persistence of the recent 11 year solar cycle 23 minimum and the relatively low cycle 24 maximum may be indicative of the onset of a grand solar minimum [*Owens et al.*, 2011; *Lockwood*, 2011]. However, the time scale and amplitude of such a grand solar minimum are unpredictable and highly uncertain. *Barnard et al.* [2011] used the *ϕ* record to construct a range of possible future scenarios for solar activity based on past variations [see also *Lockwood*, 2010]. They deduced that there is an ∼8% chance of returning to the MM‐like levels of solar activity within the next ∼40 years. However, the current rate of decline is larger than at any other point in the *ϕ* record, and hence, the estimated likelihood for this extreme scenario to occur has been increased to 15–20% (M. Lockwood, personal communication, 2015).

Given the fundamental role of solar energy in the climate system, a period of low solar activity may have important ramifications for the state of both the stratosphere and troposphere, and it is these aspects which are the focus of this study. It has been found, for example, that colder UK winters tend to occur more frequently during periods of low solar activity [*Lockwood et al.*, 2010]. *Jones et al.* [2012] examined the impact of a range of possible future TSI scenarios on global mean surface temperatures using a simple energy balance climate model. They found that a descent into MM‐like conditions over the next ∼70 years would only decrease global mean surface temperatures by up to ∼0.2 K, with some uncertainty depending on the assumed reconstruction of past TSI. *Feulner and Rahmstorf* [2010] reached similar conclusions about the impact on global surface temperature using an intermediate complexity model and two scenarios for a decline in TSI of 0.08% and 0.25% relative to 1950 levels. These results make clear that even a large reduction in solar output would only offset a small fraction of the projected global warming due to anthropogenic activities. This has been further emphasized by *Meehl et al.* [2013], who used a comprehensive climate model to show that a 0.25% decrease in TSI in the mid‐21st century would only offset the projected anthropogenic global warming trend by a few tenths of a degree.

In addition to considerations of the impact on global mean climate, where solar influences are thought to be small, there has been considerable interest and debate surrounding mechanisms for an amplified *regional* response to solar perturbations [see, e.g., *Gray et al.*, 2010, 2013]. These are broadly categorized as “top‐down” mechanisms, which focus on the impact of changes in ultraviolet (UV) radiation on stratospheric temperatures and ozone and associated changes in the extratropical stratospheric circulation [e.g., *Haigh*, 1996; *Soukharev and Hood*, 2006; *Frame and Gray*, 2010], which can impact on surface weather and climate via stratosphere‐troposphere dynamical coupling [e.g., *Kuroda and Kodera*, 2002; *Haigh et al.*, 2005; *Matthes et al.*, 2006; *Ineson et al.*, 2011]; and “bottom‐up” mechanisms, which focus on changes in surface heating and sensible and latent heat fluxes over the oceans and associated coupled air‐sea feedbacks [e.g., *White et al.*, 1997; *Meehl et al.*, 2008, 2009; *Misios and Schmidt*, 2012; *Scaife et al.*, 2013].

*Anet et al.* [2013] found amplified cooling over the Arctic and a warming over the North Atlantic in response to a 6 W m^−2^ (0.45%) decrease in TSI over the 21st century; the North Atlantic warming was related to a reduction in the weakening of the Atlantic meridional overturning circulation due to climate change, which may be partly related to a top‐down pathway [*Reichler et al.*, 2012]. *Meehl et al.* [2013] also found regionally dependent temperature changes in the tropical East Pacific in response to a sudden decrease in TSI, where an initial warm anomaly transitioned to a cold anomaly after around a decade. This is broadly similar to the East Pacific response to the 11 year solar cycle identified in some studies [e.g., *Meehl et al.*, 2009].

In addition to the effects of changes in solar irradiance, there has also been discussion around the possible climate impacts of changes in solar energetic particle fluxes. For example, *Seppälä et al.* [2013] analyzed reanalysis data and found a stronger Arctic polar vortex under high solar geomagnetic activity and changes in surface temperature that resemble the positive phase of the North Atlantic Oscillation (NAO) [*Seppälä et al.*, 2009]. Such effects will not be considered in this study because solar particles are not currently represented in the climate model employed here; further research is therefore required to test whether this may also play a role in climate in the case of a decline in solar activity.

In this study, we investigate the possible climate impacts of a descent into a deep grand solar minimum over the 21st century using a comprehensive stratosphere‐resolving coupled atmosphere‐ocean climate model. While some studies have focused on the surface response to a grand solar minimum‐like forcing, we provide further context by analyzing the effects on the stratosphere and their relationship to the surface changes. The focus of this study is on changes in the stratospheric and tropospheric circulations. A separate paper [*Ineson et al.*, 2015] examines the European wintertime surface response in more detail. The remainder of the paper is structured as follows: section 2 describes the model and experiments carried out, section 3 describes the results of the core solar minimum experiment, section 4 assesses the role of a top‐down mechanism for enhanced regional effects, and section 5 summarizes our findings.

## 2 Methods

### 2.1 The Global Climate Model

Experiments have been conducted using the Met Office's “high‐top” HadGEM2‐CC climate model, which is one configuration of the HadGEM2 model suite [*Martin et al.*, 2011]. The model is described in detail by *Hardiman et al.* [2012] and *Osprey et al.* [2013] and participated in the Coupled Model Intercomparison Project Phase 5 (CMIP5) [*Jones et al.*, 2011]. The model has 60 levels in the vertical domain with an upper boundary at ∼84 km and is run at N96 horizontal resolution (1.250°×1.875°). It includes orographic and nonorographic gravity wave drag schemes and simulates a realistic quasi‐biennial oscillation (QBO) [*Scaife et al.*, 2002]. The atmosphere is coupled to the Hadley Centre ocean model, which has 40 vertical layers and 1°×1° resolution (increasing in the tropics), and sea ice scheme as described by *Johns et al.* [2006]. The model also includes an interactive carbon cycle. The atmospheric model uses the *Edwards and Slingo* [1996] radiative transfer scheme, which has been updated to use the correlated *k* method for calculating transmittances [*Cusack et al.*, 1999]. In the configuration used here, the radiation code has six bands in the shortwave spectral region covering the intervals 200–320 nm, 320–690 nm (ozone only), 320–690 nm (ozone and water vapor), 690–1190 nm, 1190–2380 nm, and 2380–10000 nm. The radiation scheme also employs updates to the treatment of shortwave absorption by ozone as described by *Zhong et al.* [2008].

Each experiment consists of a three‐member ensemble run from 1 December 2005 to 1 January 2100 with atmospheric and oceanic initial conditions taken from three “historical” all‐forcings HadGEM2‐CC simulations. All experiments include time‐varying well‐mixed greenhouse gases (CO_2_, CH_4_, N_2_O, and chlorofluorocarbons) and aerosols as specified by the Representative Concentration Pathway 8.5 (RCP8.5) scenario [*Meinshausen et al.*, 2011]. This is a high greenhouse gas forcing scenario in which atmospheric CO_2_ concentrations increase from ∼380 ppm in 2005 to ∼970 ppm in 2100. HadGEM2‐CC does not include interactive chemistry, and thus, ozone is prescribed as a zonally averaged latitude‐height‐time field using the SPARC AC&C ozone data set [*Cionni et al.*, 2011]. This data set was recommended for use in CMIP5 and includes the recovery of the ozone layer over the 21st century due to declining abundances of ozone‐depleting substances and a climate change trend according to the SRES A1b greenhouse gas scenario. The original ozone data set did not include a solar cycle component for the future period, so this was added for the HadGEM2‐CC CMIP5 simulations (see section 2.3 for details). Unless otherwise stated, the figures presented in sections 3 and 4 show averages over the three‐ensemble members.

### 2.2 Specification of TSI and Spectral Solar Irradiance

**Figure 1:**
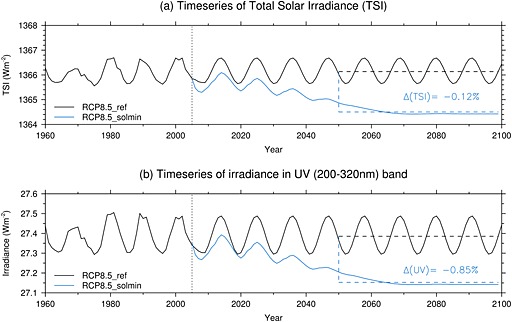
Time series of (a) TSI (W m^−2^) and (b) the irradiance in the 200–320 nm spectral band (W m^−2^) for the RCP8.5_ref (black) and RCP8.5_solmin (blue) cases. The mean percent differences for the period 2050–2099 are shown in the legends.

To explore the possible impacts of a future decline into a grand solar minimum, we use the HadGEM2‐CC RCP8.5 experiment submitted to the CMIP5 archive as a baseline (denoted RCP8.5_ref). This experiment assumes a sinusoidal 11 year solar cycle in TSI over the 21st century, with a constant amplitude based on solar cycle 23 and a fixed long‐term background (see black line in Figure 1a). The spectrally resolved irradiances are apportioned into the model's radiation bands by integrating the 1 nm fluxes provided for CMIP5 (http://solarisheppa.geomar.de/cmip5), which are derived from the Naval Research Laboratory Spectral Solar Irradiance (NRLSSI) model [*Wang et al.*, 2005]. As specified by the data set, monthly mean TSI and SSI values are used from 1882 onward and annual mean values prior to this. The irradiance in the 200–320 nm spectral band in this experiment is shown by the black line in Figure 1b. For reference, the 11 year solar max‐min change in this spectral band over the 21st century is ∼0.7% in the RCP8.5_ref experiment. We note that this represents the smallest change in solar UV irradiance indicated by the current uncertainty range [*Ermolli et al.*, 2013].

The solar perturbation experiment, denoted “RCP8.5_solmin,” includes a modified future TSI trend shown by the blue line in Figure 1a. This scenario is equivalent to the most extreme grand solar minimum case examined by *Jones et al.* [2012] (see their Figure 1) and is based on the analogue forecasts of *Barnard et al.* [2011]. The spectrally resolved irradiances for this scenario are calculated by extrapolating second‐order polynomial regressions of the irradiances in each of the six spectral bands against TSI over the period 1860–2009. The changes in TSI are thus apportioned across the spectrum using the assumption that the NRLSSI spectral data for the historical period would scale for the assumed future solar minimum scenario. The irradiance in the 200–320 nm band in this experiment is shown by the blue line in Figure 1b. The scenario corresponds to an average reduction in UV irradiance over the period 2050–2099 of 0.85% compared to RCP8.5_ref.

### 2.3 Treatment of Solar Ozone Response

Since HadGEM2‐CC does not include interactive chemistry, stratospheric and tropospheric ozone are prescribed using a modified version of the CMIP5‐recommended SPARC AC&C ozone data set [*Cionni et al.*, 2011]. The modifications include a vertical extrapolation of the ozone data above 1 hPa to coincide with the upper levels of the model domain.

The original CMIP5 ozone data set did not include a solar cycle component in the future. It has therefore been added by regressing the ozone mixing ratios at each latitude and height for the historical period onto the terms representing solar forcing ( $O_{3}^{\text{sol}}$), equivalent effective stratospheric chlorine ( $O_{3}^{\text{Cl}}$), a seasonal cycle ( $O_{3}^{\text{seas}}$), and a residual term ( $O_{3}^{\text{res}}$):

(1) $$O_{3}(t)=\alpha O_{3}^{\text{sol}}(t)+\beta O_{3}^{\text{Cl}}(t)+O_{3}^{\text{seas}}(t)+O_{3}^{\text{res}}(t)$$

The solar regression term is then added to the ozone field for the future period. A cosine latitude extrapolation of the solar term is also made over high latitudes since the signal in the original data set only extended to ±60° latitude. The magnitude of the solar max‐min ozone response in the RCP8.5_ref experiment is ∼4% in the tropical upper stratosphere. Although this magnitude is toward the upper end of estimates from observations, it is still within the plausible range [*Gray et al.*, 2009, 2010].

**Figure 2:**
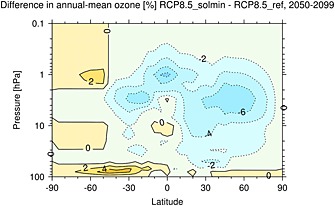
The percent differences in ozone between the RCP8.5_solmin and the RCP8.5_ref experiments for the period 2050–2099. The changes are based on the CMIP5‐recommended ozone database described by *Cionni et al.* [2011] [see also *Schmidt et al.*, 2013]. No changes are imposed at pressures higher than ∼100 hPa.

The solar ozone response term is also included in the RCP8.5_solmin experiment, with the magnitude adjusted to account for the modified future TSI trend. RCP8.5_solmin therefore includes a representation of the ozone response to solar variability [e.g., *Haigh*, 1994], which amounts to a decrease in ozone at the tropical stratopause of ∼6% for the period 2050–2099 (see Figure 2).

A summary of the experimental setups is provided in Table 1. For reference, Table 2 gives the differences in shortwave irradiances in the six spectral bands over the period 2050–2099. The analysis in section 3 focuses on the differences between RCP8.5_solmin and RCP8.5_ref for this period, and unless otherwise stated, significance testing is carried out using a two‐sided Student's *t* test for 3 × 50 years = 150 years under the assumption that each data point (e.g., a detrended monthly or seasonal mean) can be considered as an independent sample.

**Table 1:** Details of the Solar Irradiance and Prescribed Ozone Fields Used in the Experiments Described in This Study

Experiment	Solar Irradiance	Prescribed Ozone
RCP8.5_ref	Assumes a constant amplitude sinusoidal 11 year	CMIP5‐recommended SPARC
	solar cycle over the 21st century. Following CMIP5	ozone data set [*Cionni et al.*, 2011]
	recommendations, SSI is specified according to *Wang et al.* [2005].	with solar cycle regression term
		included [see *Osprey et al.*, 2013].
RCP8.5_solmin	Same as in RCP8.5_ref but assumes a large (∼0.12%)	Same as in RCP8.5_ref but
	transient decrease in TSI over the 21st century which is	solar cycle regression term altered
	distributed across the model's six spectral bands.	to be consistent with assumed
		future TSI trend.

**Table 2:** Differences in Irradiances for the Six HadGEM2‐CC Radiation Bands Averaged Over the Period 2050–2099^a^

		320–690 nm	320–690 nm				
	200–320 nm	(Ozone Only)	(Ozone and Water Vapor)	690–1190 nm	1190–2380 nm	2380–10000 nm	TSI
RCP8.5_solmin minus							
	−0.23	−0.39	−0.30	−0.42	−0.24	−0.05	−1.63
RCP8.5_ref							

a Units are in W m^−2^.

In section 3.3.2, the location of the midlatitude jet is identified by a spline interpolation of the seasonal mean zonal mean zonal wind ( ū) onto a 0.1° latitude grid and locating the maximum wind speed at 850 hPa between 30 and 70°. Jet shifts are then computed as the differences between the latitudes of the ū maxima. The changes in Southern Annular Mode (SAM) index in the same section are measured as the difference in zonal mean mean sea level pressure (MSLP) between 40–60°S and 70–90°S.

## 3 Results

### 3.1 Temperature Changes

**Figure 3:**
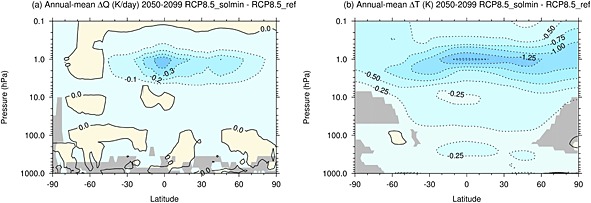
The difference in (a) annual mean zonal mean shortwave heating rates (K d^−1^) and (b) annual mean zonal mean temperature ( $\bar{T}$) between the RCP8.5_solmin and RCP8.5_ref experiments for the period 2050–2099. Grey shading indicates where the differences are not statistically significant at the 95% confidence level. The contour interval is 0.1 K d^−1^ and 0.25 K, respectively.

Figure 3a shows the differences in annual mean zonal mean shortwave heating rates (K d^−1^) between the RCP8.5_solmin and RCP8.5_ref experiments for the period 2050–2099. The grey shading indicates where the differences are not statistically significant at the 95% confidence level. At the tropical stratopause, the decrease in shortwave heating rates has a peak of ∼−0.4 K d^−1^. This localized minimum is partly related to the structure of the imposed ozone changes, which have a peak near the tropical stratopause (see Figure 2). The magnitude of the decrease in heating rate drops off rapidly with increasing latitude and decreasing altitude. There is some hemispheric asymmetry in the heating rate anomalies, with larger changes found in the Northern Hemisphere, and also some small localized increases in heating, both of which are also related to the structure of the imposed ozone changes (see Figure 2).

Figure 3b shows the corresponding differences in annual mean zonal mean temperature ( $\bar{T}$; K). The changes in temperature are closely related to the shortwave heating rate response shown in Figure 3a. There is cooling across most of the stratosphere and mesosphere which peaks at ∼1.5 K near the tropical stratopause. This can be compared to the stratospheric cooling due to climate change in the RCP8.5_ref experiment of ∼18 K at 1 hPa (2060–2099 versus 1960–1999). The upper stratospheric cooling is comparable to the solar max‐min temperature change found by *Frame and Gray* [2010], despite the fact that our TSI perturbation is approximately 1.5 times the typical amplitude of the 11 year solar cycle. However, a recent study by *Mitchell et al.* [2014] has found differences in the detailed magnitude and structure of the upper stratospheric temperature response to the 11 year solar cycle across multiple reanalysis data sets. There is also the suggestion of a weak secondary temperature maximum in the tropical lower stratosphere, similar to that identified in several reanalysis data sets [*Crooks and Gray*, 2005; *Frame and Gray*, 2010; *Mitchell et al.*, 2014]. *Gray et al.* [2009] suggested that the 11 year cycle in ozone may be an important factor in determining the structure of the temperature response in the tropical lower stratosphere. This would appear to be consistent with the imposed changes in ozone, which include a decrease in tropical lower stratospheric ozone. However, there are substantial uncertainties in estimates of the structure and amplitude of the lower stratospheric temperature signal because this region is strongly influenced by QBO variability and volcanic eruptions and the observational record is not long enough to adequately separate the signals [*Chiodo et al.*, 2014].

**Figure 4:**
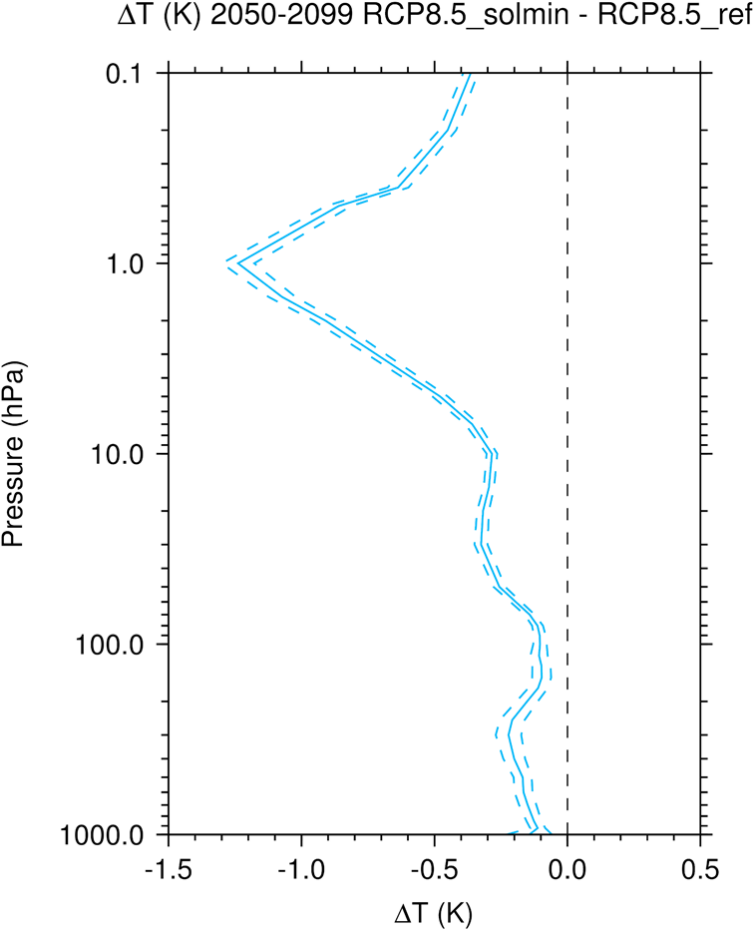
The difference in global mean annual mean $\bar{T}$ (K) between the RCP8.5_solmin and RCP8.5_ref experiments for the period 2050–2099. The dashed lines show the 95% confidence intervals.

Figure 4 shows the vertical profile of differences in annual and global mean $\bar{T}$. The maximum cooling occurs at 1 hPa with a magnitude of 1.2 K and decreases rapidly in magnitude above and below this level. From 10 to 50 hPa the cooling is roughly constant in height with a magnitude of ∼0.3 K. There is cooling throughout the troposphere, which increases with altitude from ∼0.1 K at the surface to ∼0.25 K near the tropopause. The change in global mean 1.5 m temperature ( ${\bar{T}}_{1.5m}$) for the period 2050–2099 is −0.13 K. This is broadly consistent with the energy balance model results of *Jones et al.* [2012].

The results in this section show that evolving into a grand solar minimum over the 21st century has the potential to enhance stratospheric cooling trends due to increasing carbon dioxide concentrations, but as has been highlighted in other recent studies [*Jones et al.*, 2011; *Meehl et al.*, 2013], such a decline would have only a small impact on any anthropogenic global warming trend.

### 3.2 Stratospheric Changes

A solar cycle influence on the high‐latitude stratosphere has been identified in reanalysis data and climate models [e.g., *Kuroda and Kodera*, 2002; *Matthes et al.*, 2006; *Ineson et al.*, 2011; *Mitchell et al.*, 2014]. Given the interhemispheric differences in the generation of planetary wave activity in the troposphere, which partly determines the mean strength and unforced variability of the winter polar vortices, it is perhaps unsurprising that the dynamical responses to external forcings, such as the QBO and solar variability, tend to be different in the two hemispheres [e.g., *Anstey and Shepherd*, 2014]. In the Northern Hemisphere (NH), studies have shown a time‐averaged solar cycle signal in the high‐latitude stratosphere consisting of a poleward and downward propagation of zonal wind and temperature anomalies over the winter season [*Kuroda and Kodera*, 2002]. The main mechanism proposed to explain the propagation and amplification of these anomalies invokes wave‐mean‐flow interactions [e.g., *Kodera et al.*, 2003; *Ineson et al.*, 2011]. In contrast, the extratropical circulation response to an external forcing in the Southern Hemisphere is often manifested around the time of the spring breakup of the polar vortex [e.g., *Kuroda and Kodera*, 2005]. We now discuss the stratospheric circulation response to the imposed decline in solar activity.

#### 3.2.1 Northern Hemisphere

**Figure 5:**
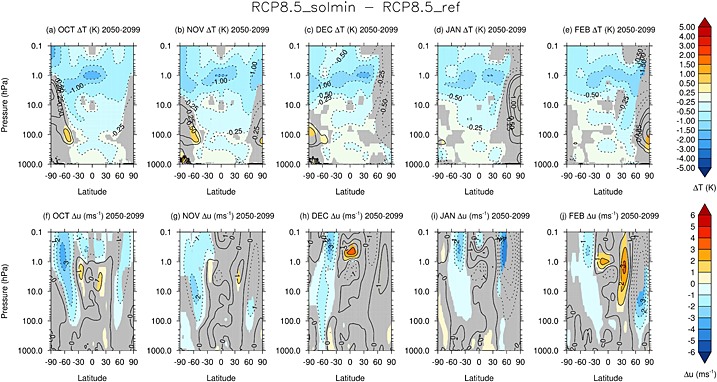
The difference in monthly mean (a–e) $\bar{T}$ (K) and (f–j) ū (m s^−1^) for the period 2050–2099 between the RCP8.5_solmin and RCP8.5_ref experiments. The panels show data for October–February, respectively. The grey shading denotes regions where the differences are not statistically significant at the 95% confidence level.

Figures 5a–5e show monthly mean $\bar{T}$ differences between the RCP8.5_solmin and RCP8.5_ref experiments for October to February averaged over the period 2050–2099. The shading is as in Figure 3. In the NH, there is a relative warming of the Arctic lower stratosphere in February. Since the direct radiative tendency of the decrease in solar irradiance would be to cool the stratosphere, the high‐latitude warming is indicative of a dynamical response to the solar perturbation. Further analysis (not shown) shows that there is an increase in wave driving (i.e., Eliassen Palm flux divergence) in the high‐latitude middle and lower stratosphere [cf. *Ineson et al.*, 2011], particularly in January, and the associated dynamical heating acts against the radiatively driven cooling.

Figures 5f–5j show equivalent plots to Figures 5a–5e for differences in zonal mean zonal wind. The warming of the Arctic polar vortex in boreal winter is coincident with a weakening of the stratospheric westerly jet. There is an easterly anomaly in the region of the jet core (∼1 hPa) of up to 3–4 m s^−1^ in January–February. A weaker easterly anomaly, more confined to the middle and upper stratosphere and the mesosphere, is also present in October–November, but the differences in December are not highly statistically significant, probably in part due to the large interannual variability during NH midwinter.

**Figure 6:**
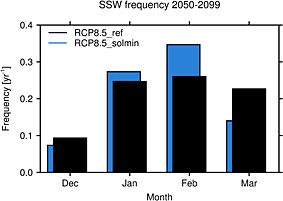
The wintertime distribution of Arctic stratospheric sudden warming events (year^−1^) in the RCP8.5_ref (black) and RCP8.5_solmin (blue) experiments for the period 2050–2099.

It has been suggested that changes in solar irradiance may impact on the timing of major sudden stratospheric warming events (SSWs) [see, e.g., *Gray et al.*, 2004], which occur in the Arctic stratosphere during boreal winter. Thus, some of the changes in ū in Figures 5f–5j may reflect changes in the frequency or timing of SSWs. Figure 6 shows the wintertime distribution of SSWs in each experiment for the period 2050–2099 using the *Charlton and Polvani* [2007] definition based on a temporary reversal of ū at 10 hPa and 60°N to easterlies. The results for the RCP8.5_ref experiment have been previously discussed by *Mitchell et al.* [2012]. The histograms suggest that although the average number of SSWs in RCP8.5_solmin remains similar to that in RCP8.5_ref (0.83 year^−1^), there is a slight decrease in the occurrence of SSWs in March and an increase in January–February. However, these changes are not highly statistically significant according to the *t* test formulated by *Charlton et al.* [2007], which partly reflects the large interdecadal variability in SSWs, as noted by *Butchart et al.* [2000]. Consequently, longer simulations would be required to make robust conclusions about whether a decline in solar activity would impact on the frequency or timing of SSWs.

#### 3.2.2 Southern Hemisphere

**Figure 7:**
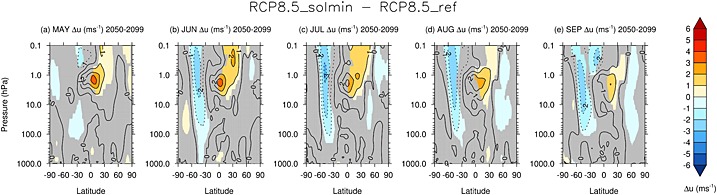
As in Figures 5f–5j but for May–September, respectively.

In the Southern Hemisphere (SH), the plots of monthly mean $\bar{T}$ in Figures 5a–5c show a relative warming of the Antarctic lower stratosphere by up to ∼1 K during the SH dynamically active season (October–December). This reflects a decrease in the equator‐to‐pole temperature gradient and is coincident with a weakening of the climatological westerly jet throughout the stratosphere by up to ∼3 m s^−1^ (Figures 5f–5h). In midwinter (June, July, and August; JJA), there is an easterly anomaly in the midlatitudes (30–60°S) of up to ∼3 m s^−1^ (see Figure 7), which reflects a weakening of the westerlies on the equatorward flank of the stratospheric jet. These changes in circulation which extend throughout the stratosphere are in contrast to the response in austral summer (January–February), where the subtropical easterly anomaly is mainly confined to the upper stratosphere and mesosphere.

### 3.3 Tropospheric Changes

Circulation changes in the stratosphere during the dynamically active seasons, such as those described in section 3.2, can impact on the underlying troposphere via stratosphere‐troposphere dynamical coupling (see, e.g., *Gerber et al.* [2012] for an overview). Furthermore, a number of studies have shown a potential influence of solar variability on the tropical Pacific Ocean and the El Niño–Southern Oscillation (ENSO) [e.g., *Meehl et al.*, 2009]. We now discuss the changes in the tropospheric state in the grand solar minimum experiment.

#### 3.3.1 Northern Hemisphere

**Figure 8:**
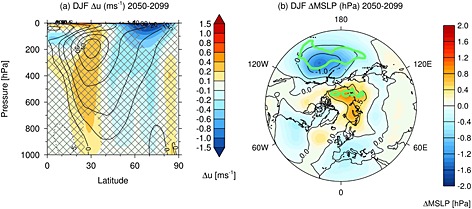
The differences in seasonal mean DJF (a) ū (m s^−1^) and (b) MSLP (hPa) for the period 2050–2099 between the RCP8.5_solmin and RCP8.5_ref experiments. Note that the shading intervals in Figure 8a are not constant and that data are only shown for the Northern Hemisphere. The solid contours in Figure 8a denote the 2050–2099 ū climatology in the RCP8.5_ref experiment and are marked at ±5, 10, 20, 30, 40, and 50 m s^−1^. The hatching in Figure 8a denotes regions where the differences are not statistically significant at the 95% confidence level. The green lines in Figure 8b encompass regions where the differences are statistically significant at the 95% confidence level.

Figure 8a shows the seasonal mean tropospheric ū changes in the NH in December, January, and February (DJF). The shading denotes the differences between the RCP8.5_solmin and RCP8.5_ref experiments, and the contours show the climatology of the latter for reference. The hatching denotes where the differences are not statistically significant at the 95% confidence level. There is a barotropic dipole change in ū in the region of the midlatitude jet, with a westerly anomaly between 30 and 45°N and an easterly anomaly between 50 and 70°N. This feature shows a peak‐to‐peak ū dipole change of 0.36 m s^−1^ at 850 hPa. The dipole ū response is comparable to the climate change signal in DJF in the NH (2060–2099 versus 1960–1999), which shows a strengthening of the westerlies in the jet core by 0.5 m s^−1^ and a weakening of the westerlies on the poleward flank by ∼0.9 m s^−1^ (not shown). Thus, while the impact of the decline in solar activity on global near‐surface temperature is relatively small, its effects on the midlatitude circulation amount to a considerable fraction of the uncertainty due to future greenhouse gas trends [see also *Ineson et al.*, 2015]. A significant NH tropospheric ū response is not found outside of boreal winter, which suggests a role for a top‐down influence of changes in the stratospheric circulation on middle‐ and high‐latitude climate.

Figure 8b shows a polar stereographic map of the differences in DJF MSLP (hPa) between the RCP8.5_solmin and RCP8.5_ref experiments. The green lines encompass regions where the differences are statistically significant at the 95% confidence level. A more negative Arctic Oscillation (AO) index is characterized by lower pressure in the midlatitudes and higher pressure over the polar cap, which corresponds to a weakening of the climatological equator‐to‐pole pressure gradient and anomalously easterly flow across Europe and the Atlantic sector [*Thompson and Wallace*, 1998]. The pattern in Figure 8b suggests a more negative AO index, although the response is not highly statistically significant and the structure over the North Atlantic does not strongly resemble the NAO. There is a deepening of the Aleutian Low, which has also been identified during solar minimum conditions in observations [e.g., *Roy and Haigh*, 2010; *Gray et al.*, 2013].

Blocking episodes have been highlighted as an important aspect of variability in the North Atlantic circulation [e.g., *Shabbar et al.*, 2001; *Woollings et al.*, 2010b]. Previous studies have identified variations in blocking frequency associated with the 11 year solar cycle [*Barriopedro et al.*, 2008; *Woollings et al.*, 2010a]. The solar‐blocking signal identified in these studies consists of an increase in Euro‐Atlantic blocking during solar minimum, with the precise magnitude of the changes being somewhat sensitive to the metric used to define solar activity (e.g., F10.7 cm radio flux or open solar flux), but is typically around ∼8–10% of total blocked days.

**Figure 9:**
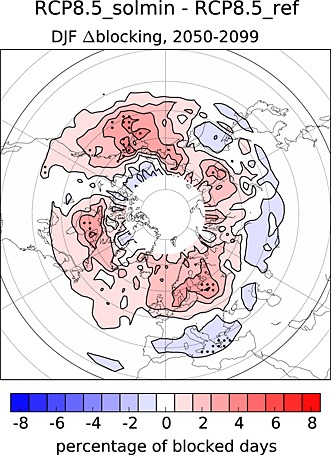
The difference in ensemble mean DJF blocking frequency (as a percent of blocked days) between the RCP8.5_solmin and RCP8.5_ref experiments for the period 2050–2099. Blocking events are defined using a metric based on potential temperature on the dynamical tropopause and is consistent with that used by *Woollings et al.* [2010a]. The stippling indicates where the differences are significant at the 95% confidence level.

Figure 9 shows differences in the ensemble mean DJF blocking frequency (as a percent of total blocked days) between the RCP8.5_solmin and RCP8.5_ref experiments. The blocking index used here is based on temporary reversals in the meridional gradient of potential temperature on the dynamical tropopause which must persist for at least 5 days and is identical to that used by *Woollings et al.* [2010a] and *Anstey et al.* [2013]. The general pattern of an increase in Euro‐Atlantic and Pacific blocking at high latitudes and a decrease over the Mediterranean are consistent with the results of previous studies [e.g., *Woollings et al.*, 2010a], but the magnitudes of the differences are several times smaller. Like many CMIP5 models, HadGEM2‐CC has biases in its representation of NH blocking, the main features of which are a lack of blocking events at high latitudes and too much blocking at lower latitudes [*Anstey et al.*, 2013]. It is possible that the underlying model biases could impact on the simulation of a solar‐blocking connection [e.g., *Scaife et al.*, 2011]. However, the reanalysis‐based studies described above have mostly focused on the late twentieth century period, when there was an *immediate* correlation between solar variability and the NAO (and, by proxy, blocking events), but the solar‐NAO relationship at *zero lag* has been shown to be considerably weaker over a longer record [*Roy and Haigh*, 2010]. *Gray et al.* [2013] showed that over the longer period 1870–2010 the strongest correlation was at lags of 3–4 years, but the HadGEM2‐CC model was unable to reproduce this behavior, leading *Scaife et al.* [2013] to suggest that there may be deficiencies in the representation of midlatitude ocean‐atmosphere coupling in the model. Despite these outstanding questions, our results are consistent with the findings of other studies which have highlighted a solar influence on NH blocking and the NAO.

The results in this section show that there is a coherent change in the NH extratropical circulation in response to the decline in solar activity which extends from the upper stratosphere to the surface [cf., e.g., *Ineson et al.*, 2011; *Gray et al.*, 2013].

#### 3.3.2 Southern Hemisphere

**Figure 10:**
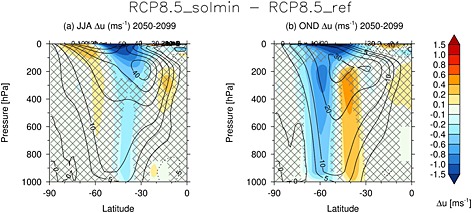
As in Figure 8a but for the Southern Hemisphere (SH) for (a) JJA and (b) OND seasons. The contours denote the 2050–2099 climatology in the RCP8.5_ref experiment. The hatching denotes regions where differences are not statistically significant at the 95% confidence level.

As was shown in Figure 5, the SH high‐latitude zonal wind anomalies in December extend throughout the stratosphere and are accompanied by dipole changes in the troposphere in the region of the midlatitude jet. Figure 10 shows differences in the seasonal mean ū between the RCP8.5_solmin and RCP8.5_ref experiments for JJA (a) and October, November, and December (OND) (b) seasons. In JJA, there is a small poleward shift in the midlatitude jet, with a peak‐to‐peak dipole change in ū at 850 hPa of 0.32 m s^−1^. The strongest signal is a weakening of the westerlies on the equatorward flank of the jet. In OND, there is an equatorward jet shift of ∼0.5° latitude, with a peak‐to‐peak ū dipole of 0.87 m s^−1^.

**Figure 11:**
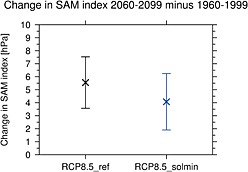
The difference in SAM index (hPa) between 2060–2099 and 1960–1999 for the RCP8.5 and RCP8.5_solmin experiments compared to the historical experiment. The SAM index is defined as the difference in zonally averaged MSLP between 40–60°S and 70–90°S. The whiskers show 5–95% confidence intervals.

Projected future trends in the position of the SH midlatitude jet have been shown to be sensitive to the recovery of the Antarctic ozone hole and increases in greenhouse gas concentrations [*Son et al.*, 2008]. In austral winter, the trend in jet position is largely determined by the greenhouse gas forcing [*Barnes et al.*, 2014]. In the baseline RCP8.5 experiment, there is a poleward shift in the jet of 2.5° ( $2.7\;m\;s^{-1}ū$ dipole) in JJA and 2.0° ( $3.5\;m\;s^{-1}ū$ dipole) in OND (2060–2099 versus 1960–1999). The change in jet latitude in OND is coincident with a more negative Southern Annular Mode (SAM) index of ∼1.4 hPa, which offsets the positive SAM trend in this season in the RCP8.5_ref experiment by ∼25% (see Figure 11).

Interestingly, the circulation changes in JJA are of the opposite sign to what is typically associated with stratosphere‐troposphere dynamical coupling (weaker stratospheric westerlies lead to a more equatorward tropospheric jet). Such a seasonal dependence of the sign of the SH jet shift has been identified in other studies. *Varma et al.* [2011] found that the response to a constant 2 W m^−2^ reduction in TSI consisted of a poleward jet shift in JJA and an equatorward jet shift in DJF in a coupled model without a well‐resolved stratosphere. The annual mean response was dominated by the signal in DJF (i.e., an equatorward jet shift). *Thresher* [2002] found observational evidence for a seasonal cycle in the SH surface solar response over the late twentieth century, which would be consistent with the findings of *Varma et al.* [2011]. However, the findings of modeling studies may be sensitive to, e.g., the inclusion of a well‐resolved stratosphere, and thus, this highlights the need for further research to better understand the response of the SH circulation to stratospheric changes and its dependence on season.

#### 3.3.3 Tropics

In addition to the proposed top‐down mechanisms for an amplified surface response to solar forcing in the midlatitudes, some studies have proposed an additional bottom‐up mechanism operating in the tropical Pacific. This involves coupled air‐sea feedbacks in response to small changes in surface heating and results in an anomalous sea surface temperature (SST) pattern that resembles ENSO [*White et al.*, 1997, *Meehl et al.*, 2008, 2009]. However, there has been some disagreement as to whether the observed response corresponds to a warm or cold ENSO phase at solar maximum, and *Roy and Haigh* [2010] further showed that the apparent solar‐ENSO connection could be due to aliasing onto unconnected ENSO variations.

**Figure 12:**
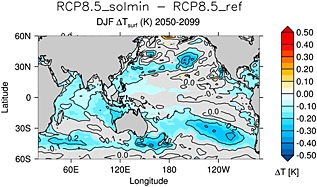
The difference in DJF sea surface temperature (K) for the period 2050–2099 between the RCP8.5_solmin and RCP8.5_ref experiments. The solid black contours denote 0.1 K intervals. The light grey shading denotes regions that are not statistically significant at the 95% confidence level.

As described in section 1, *Meehl et al.* [2013] identified an ENSO‐like response in model simulations of a persistent solar minimum, with relatively warm East Pacific SSTs during the first decade after a reduction in TSI was imposed, followed by colder SSTs in the second decade. However, the interdecadal changes were not found to be highly statistically significant. Figure 12 shows the differences in DJF sea surface temperatures between the RCP8.5_solmin and RCP8.5_ref experiments. There is weak cooling (∼0.1 K) across much of the tropical Pacific; the change in area‐averaged (15°N–15°S, 150°E–90°W) temperature is −0.075 K. However, there is no indication of a local amplification in the ENSO region. Our simulations therefore do not lend support to the existence of a solar‐ENSO connection. This is in contrast to the results of *Meehl et al.* [2013], although their simulated ENSO‐like response to a persistent solar minimum was weaker than that found for 11 year cycle variations [*Meehl et al.*, 2009]. However, the experiments do show enhanced cooling over the North Pacific, which is consistent with the deepened Aleutian Low [e.g., *Schneider and Cornuelle*, 2005]. There is also a band of stronger cooling across the SH midlatitudes, which may be partly related to changes in the midlatitude jet (see section 3.3.2).

## 4 Sensitivity to UV Forcing

The RCP8.5_solmin experiment shows enhanced regional surface responses to a decline in solar activity, particularly in the middle and high latitudes. As described in section 1, one proposed mechanism for such localized effects involves the impact of changes in shortwave heating rates on the stratospheric circulation and subsequent surface impacts via stratosphere‐troposphere coupling. The potential for this mechanism to contribute to the response to solar forcing in the Northern Hemisphere was demonstrated by *Ineson et al.* [2011]. They imposed a perturbation in 200–320 nm radiation (i.e., in UV radiation alone) in a model and found a more negative NAO index under solar minimum conditions.

To identify whether a similar mechanism may also be operating here, we conduct a further experiment (RCP8.5_uvmin) in which the 200–320 nm irradiance is reduced by 6.4% in isolation of any other changes (e.g., ozone and visible radiation); this enables a separation of a pure top‐down influence from a decline in solar activity. This is a highly idealized experiment, in which the imposed UV perturbation is considerably larger than in RCP8.5_solmin, and other effects, such as the solar ozone response, are neglected. Nevertheless, it allows us to make an assessment of at least one pathway that may be contributing to the RCP8.5_solmin results discussed in the previous sections and to elucidate more generally the role of the top‐down pathway for solar‐climate coupling. The same experimental protocol as described in section 2 is carried out, with a reduction in 200–320 nm radiation (and by definition in TSI) of 1.75 W m^−2^ over the 2050–2099 period.

**Figure 13:**
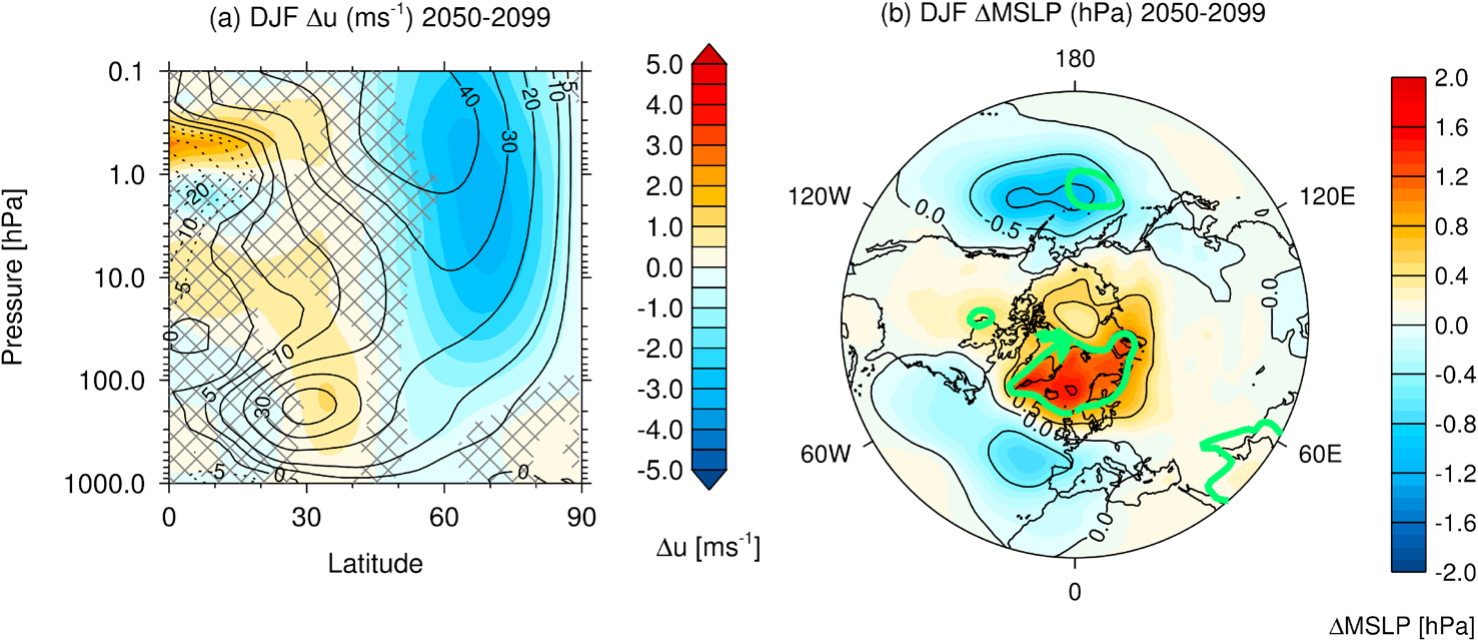
As in Figure 8 but for the differences between the RCP8.5_uvmin and RCP8.5_ref experiments. Note that data are only shown for the Northern Hemisphere.

Figure 13a shows the December–February mean difference in ū between the RCP8.5_uvmin and the RCP8.5_ref experiments over 2050–2099. There is a weakening of the stratospheric jet by up to 3 m s^−1^ and poleward shift in the tropospheric jet. This is qualitatively similar to the response in the RCP8.5_solmin experiment (Figures 5 and 8), but about 20–30% larger, and is consistent with a top‐down pathway which contributes to the amplified regional climate responses.

Figure 13b shows the DJF mean sea level pressure differences between the RCP8.5_uvmin and RCP8.5_ref experiments. The changes in tropospheric ū are commensurate with a more negative NAO index and a deepening of the Aleutian Low, again with the amplitude of the changes being slightly larger than in RCP8.5_solmin. Conversely, in the SH (not shown), there is no indication of an enhanced tropospheric response during OND, as was found in RCP8.5_solmin in section 3.3.2.

These results suggest that changes in UV irradiance and a top‐down influence are likely to be contributing to the enhanced NH surface response discussed in section 3.3 but that the enhanced response in the SH in OND may be related to other processes, such as the ozone response or changes in visible irradiance. Future studies should therefore aim to elucidate the roles of these mechanisms in driving the SH response to solar forcing.

## 5 Summary and Discussion

A comprehensive coupled atmosphere‐ocean global climate model with a well‐resolved stratosphere (HadGEM2‐CC) has been used to investigate the possible impacts of evolving into a period of very low solar activity over the 21st century. The assumed scenario is akin to what may have occurred during the Maunder Minimum (MM) in the late seventeenth century. The RCP8.5_solmin experiment assumes a mean decrease in TSI of ∼0.12% over the second half of the 21st century and includes a decrease in UV irradiance (200–320 nm) of 0.85%, along with a representation of the solar cycle impact on stratospheric ozone.

The key conclusions of the study for projections of global mean climate are as follows:

1. A return to MM‐like levels of solar activity would enhance the anticipated stratospheric cooling trend due to increasing atmospheric carbon dioxide concentrations. The maximum cooling at the stratopause is ∼1.2 K, which can be compared to the projected cooling due to climate change in the RCP8.5 scenario of ∼18 K.
2. The change in global mean near‐surface temperature over the second half of the 21st century is *O*(0.1 K), confirming the findings of earlier studies which have shown that a large decrease in solar activity would do little to offset the projected anthropogenic global warming trend [cf. *Feulner and Rahmstorf*, 2010, *Jones et al.*, 2011, *Meehl et al.*, 2013, *Anet et al.*, 2013].

In the NH during boreal winter, the main features of the response to the solar minimum consist of the following:

1. A warmer polar lower stratosphere and slight weakening (<4 m s^−1^) of the polar vortex, with the largest changes occurring in January–February.
2. Dipole changes in NH ū in DJF in the region of the midlatitude jet. The changes in the large‐scale circulation suggest a more negative Arctic Oscillation index, but the pattern over the North Atlantic does not strongly resemble the NAO.
3. Changes in the occurrence of NH tropospheric blocking events, with an increase over Northern Europe and the North Pacific and a decrease over Southern Europe. The magnitude of this change is smaller than has been suggested in studies using reanalysis data for the recent past, but the patterns are similar [*Woollings et al.*, 2010a].
4. A further sensitivity experiment which only included changes in 200–320 nm (UV) radiation indicates that the enhanced NH regional responses are at least partly driven by changes in UV irradiance and a top‐down pathway.

A separate paper [*Ineson et al.*, 2015] describes the European wintertime surface response and land surface temperature changes in more detail.

In the SH during austral winter and spring, we find that the decrease in solar activity leads to the following:

1. A relative warming of the Antarctic stratosphere during June–December. This is coincident with a weakening of the background stratospheric westerly jet of up to 3 m s^−1^.
2. An equatorward shift in the Southern Hemisphere (SH) tropospheric midlatitude jet by ∼0.5° and a more negative Southern Annular Mode index of ∼1.4 hPa in October–December (OND).

Finally, in contrast to earlier studies [e.g., *Meehl et al.*, 2009], we find no evidence of an enhanced sea surface temperature response over the tropical Pacific that would be suggestive of an impact on ENSO. Our experiment therefore does not lend support to the existence of a solar‐ENSO connection.

It is projected that over the 21st century there will be significant changes in the tropospheric circulation due to the combined effects of ozone recovery and increasing greenhouse gas concentrations [e.g., *Wilcox et al.*, 2012; *Scaife et al.*, 2012]. Our experiment has shown that although any impact on global mean surface temperature can be expected to be small, uncertainties in future solar forcing should be considered in projections of regional high‐latitude climate change. It is also important to note that although some studies have presented arguments for a future decline in solar output [e.g., *Barnard et al.*, 2011; *Abreu et al.*, 2008], the CMIP5 integrations assumed no trend in solar activity in the future. It is therefore important that more scenarios which reflect the range of possible future changes in solar activity should be generated for use in studies of 21st century climate. We further emphasize that the recommended representation of spectral solar irradiance in CMIP5 was based on the *Wang et al.* [2005] data set, which is at the lower end of the estimated range for UV variability [*Ermolli et al.*, 2013]. We therefore highlight the need for alternate scenarios which better reflect the current understanding of SSI variability for use in future model intercomparisons.
